# Supplementary figures and images for: Spatial heterogeneity of soil properties in planted mixed forests in the rocky desertification areas of the Wuling Mountain
Source: PeerJ. 2024 Dec 23;12:e18724. doi: 10.7717/peerj.18724 (PMC11670765; doi:10.7717/peerj.18724)

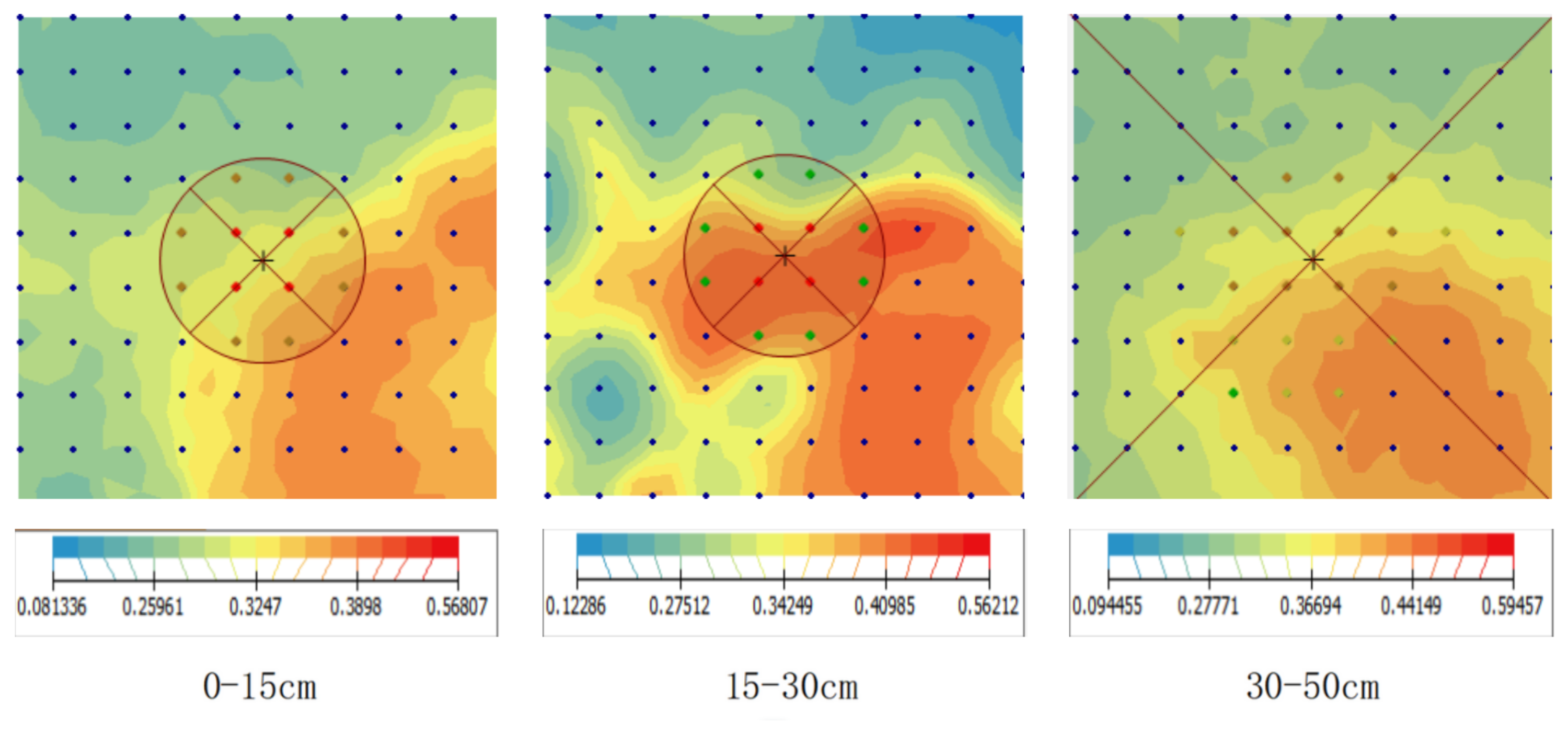

Supplement: Supplemental Information 1 [file peerj-12-18724-s001.png]

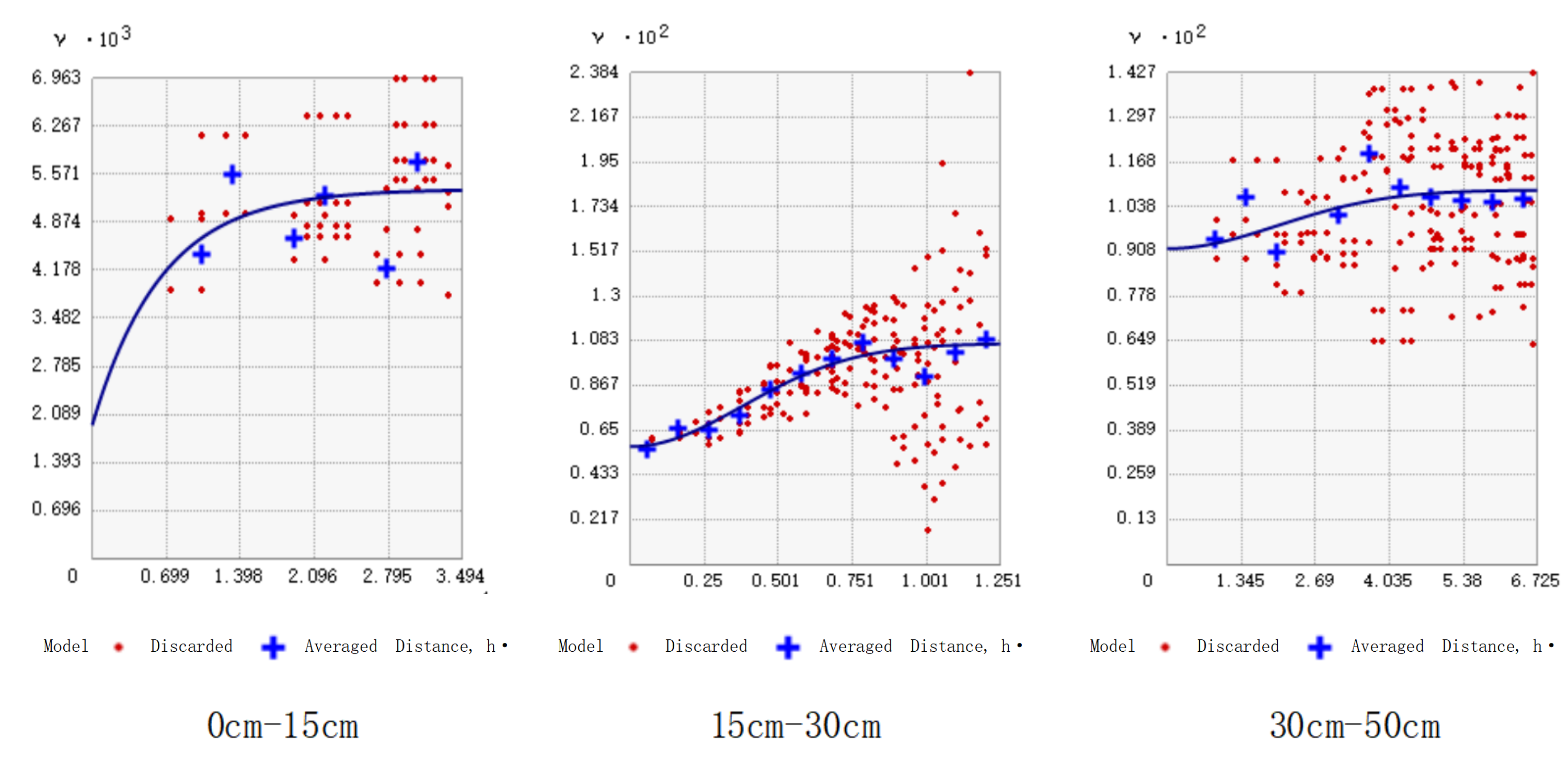

Supplement: Supplemental Information 2 [file peerj-12-18724-s002.png]

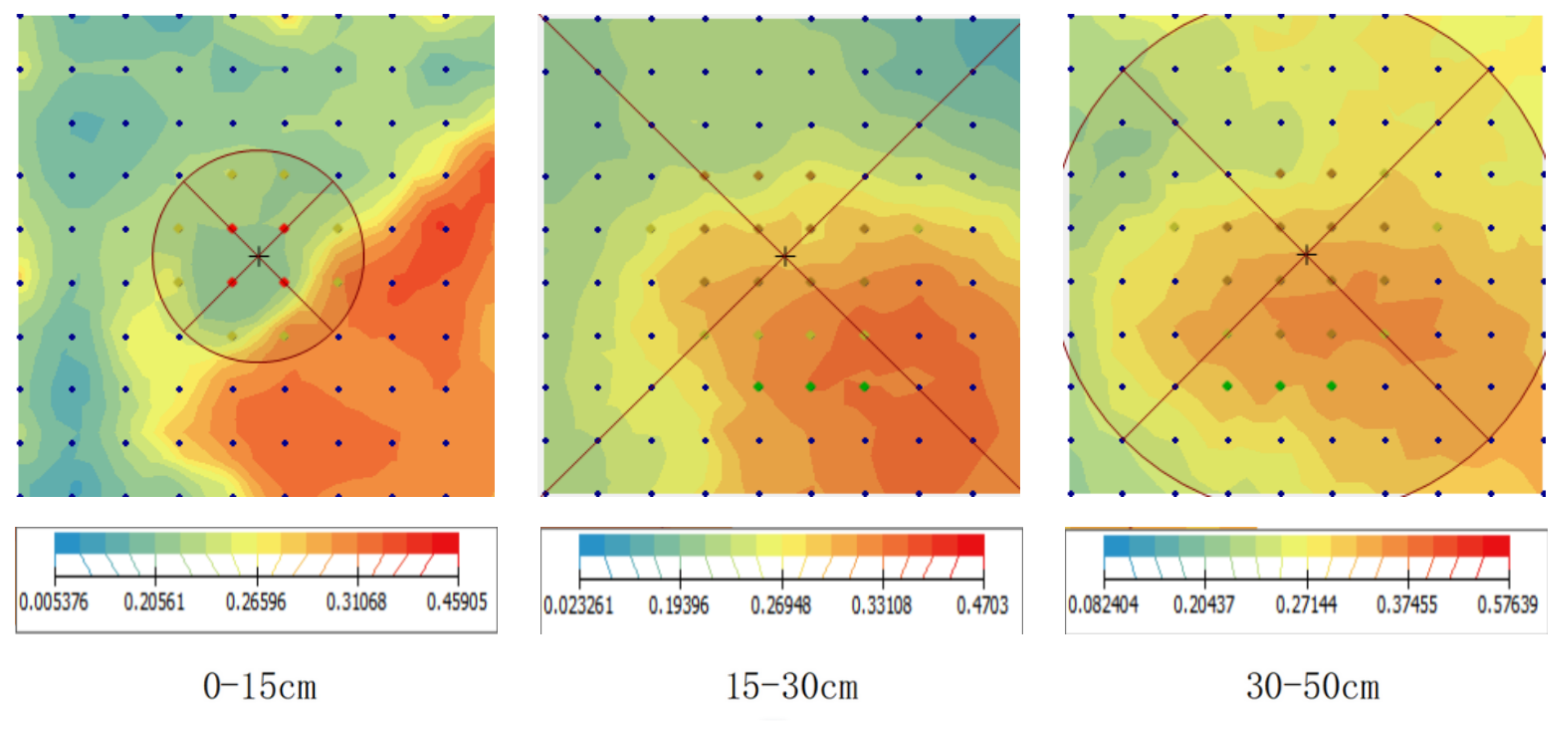

Supplement: Supplemental Information 3 [file peerj-12-18724-s003.png]

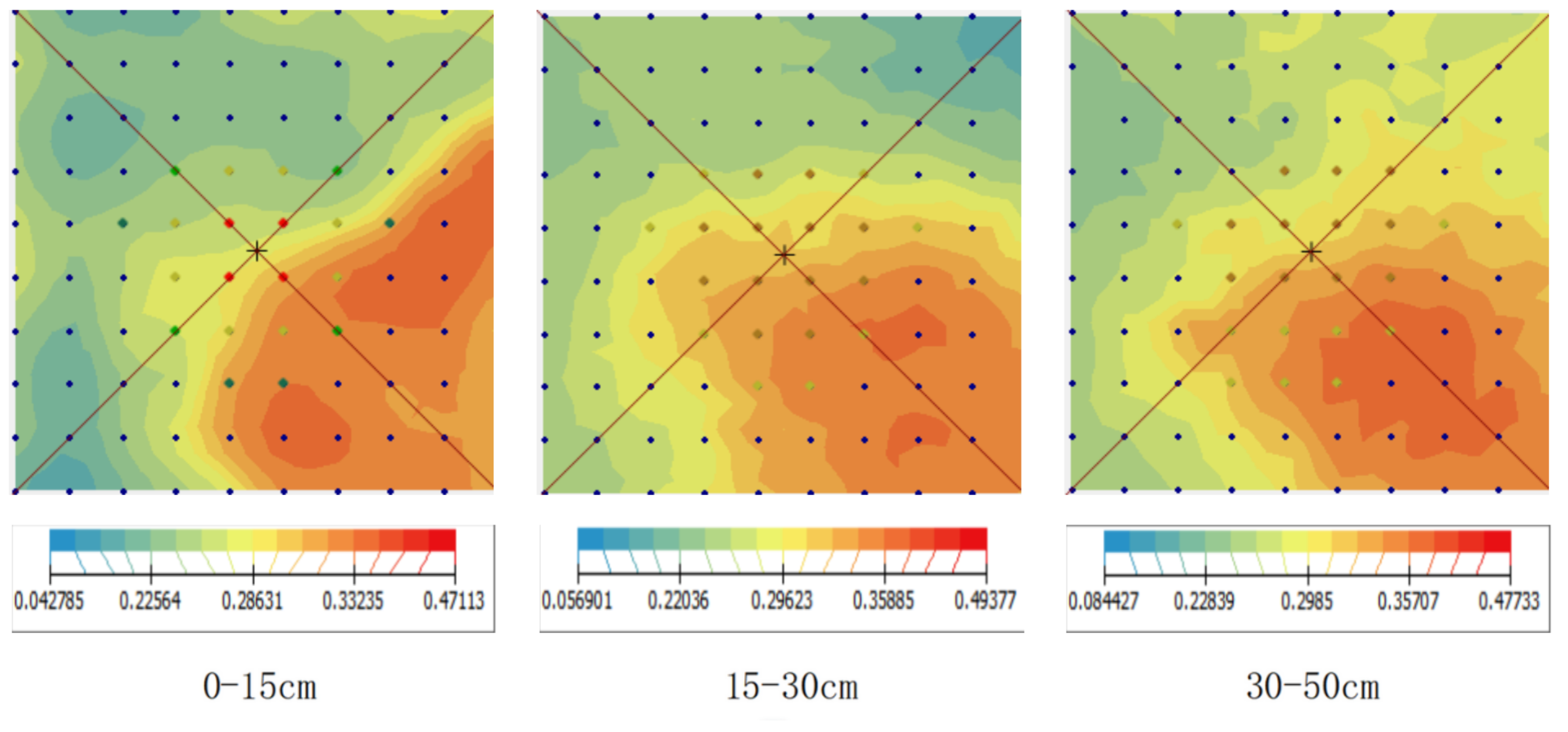

Supplement: Supplemental Information 4 [file peerj-12-18724-s004.png]

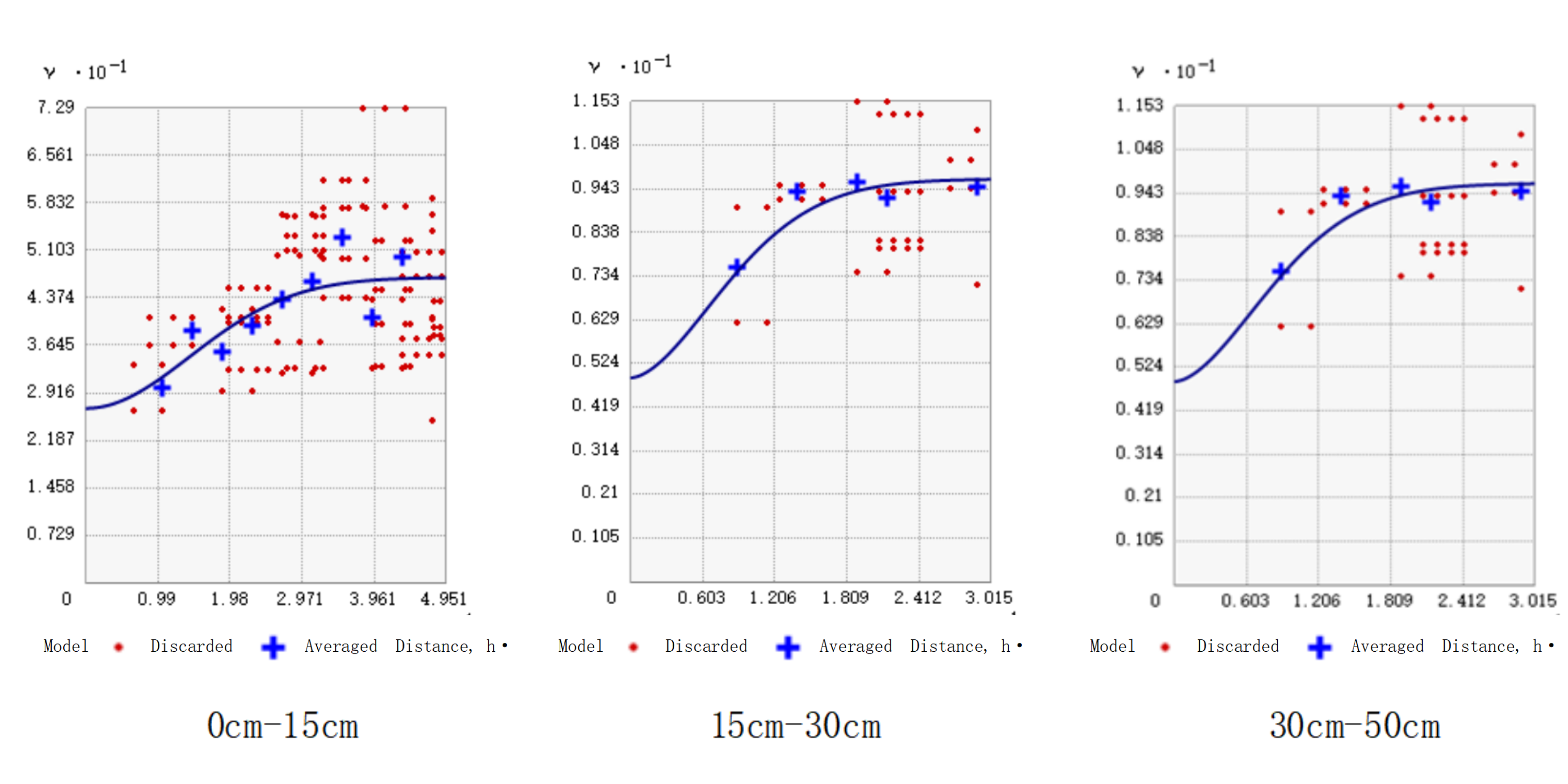

Supplement: Supplemental Information 5 [file peerj-12-18724-s005.png]

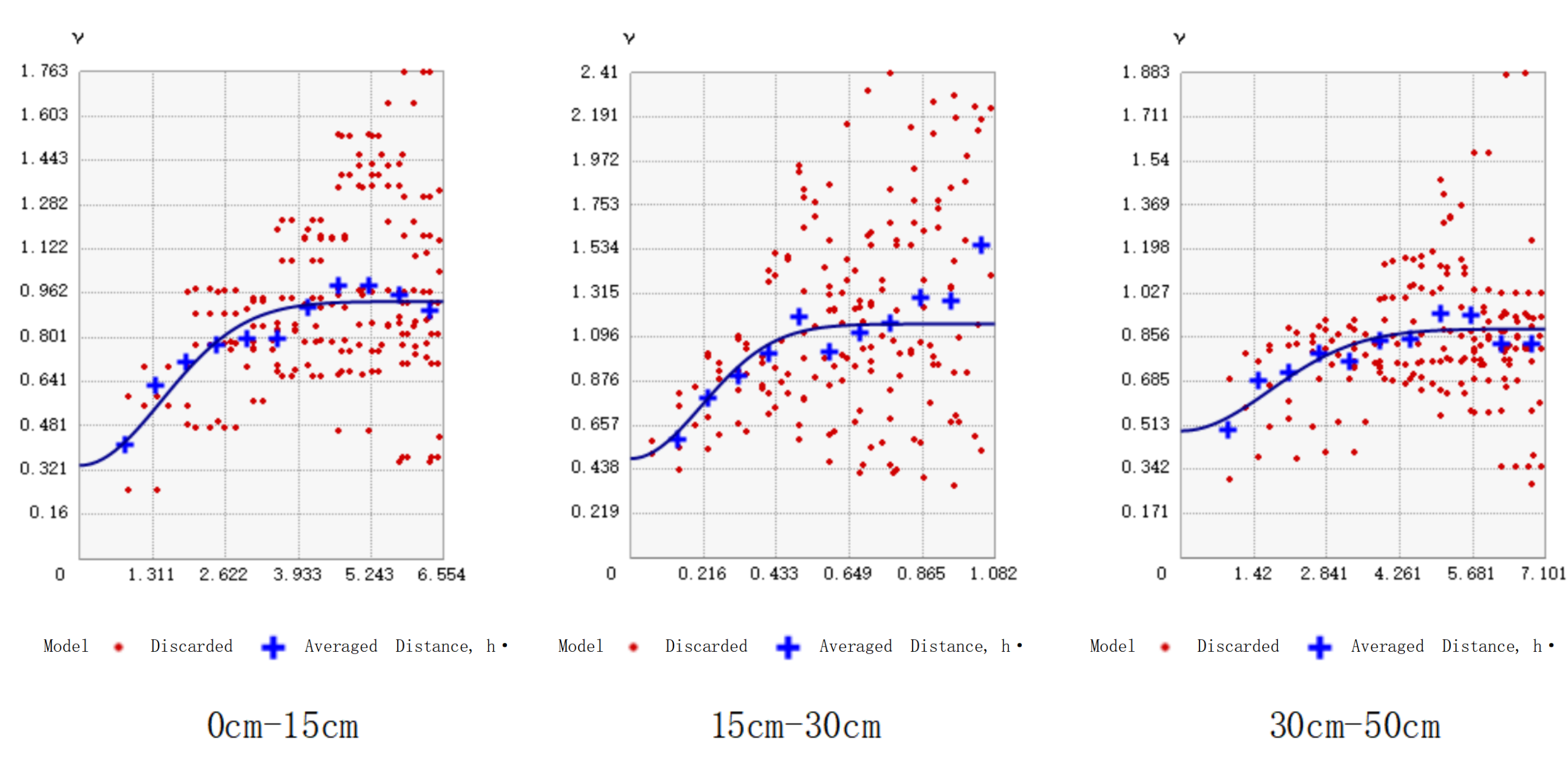

Supplement: Supplemental Information 6 [file peerj-12-18724-s006.png]

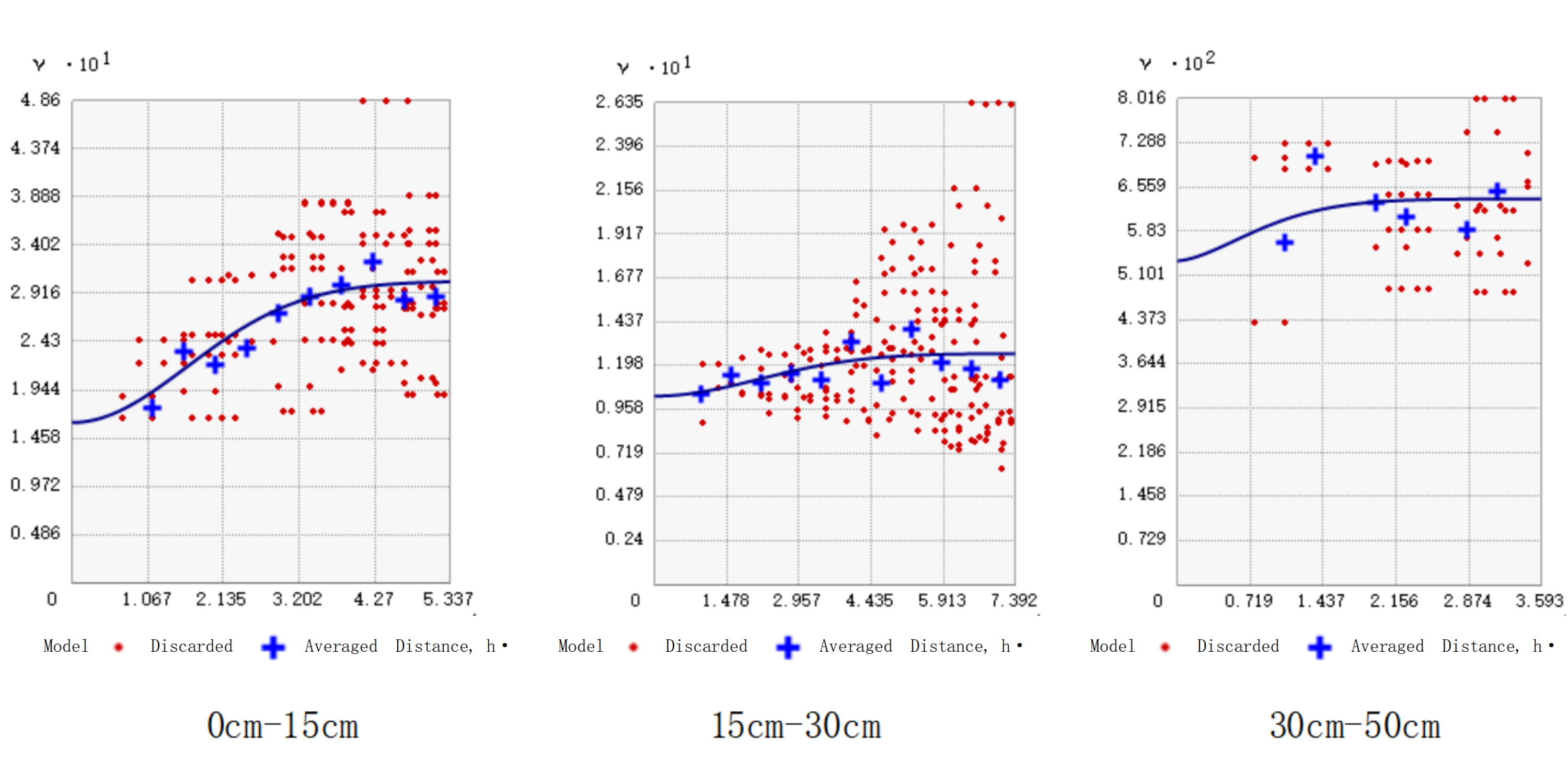

Supplement: Supplemental Information 7 [file peerj-12-18724-s007.png]

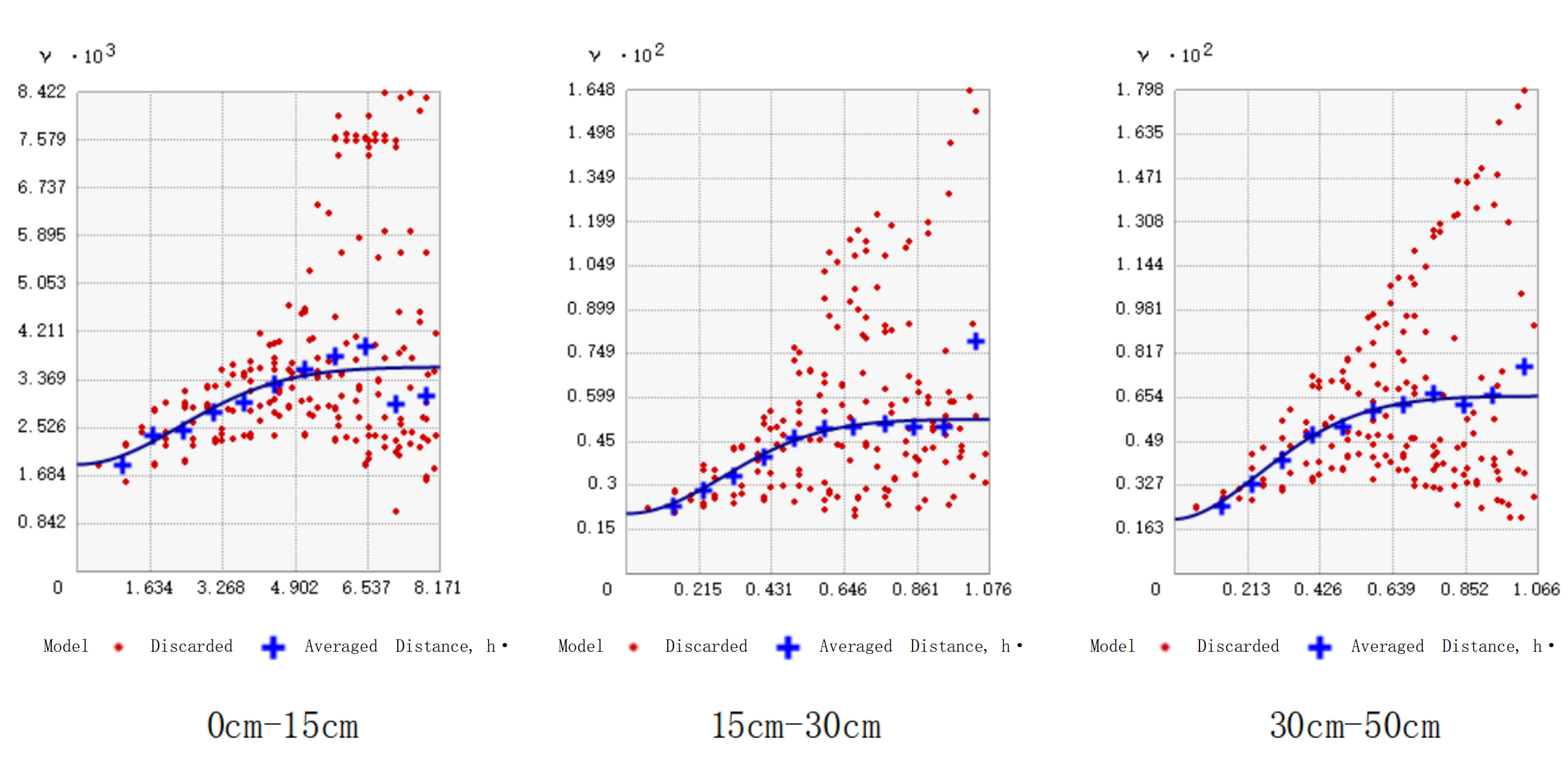

Supplement: Supplemental Information 8 [file peerj-12-18724-s008.png]

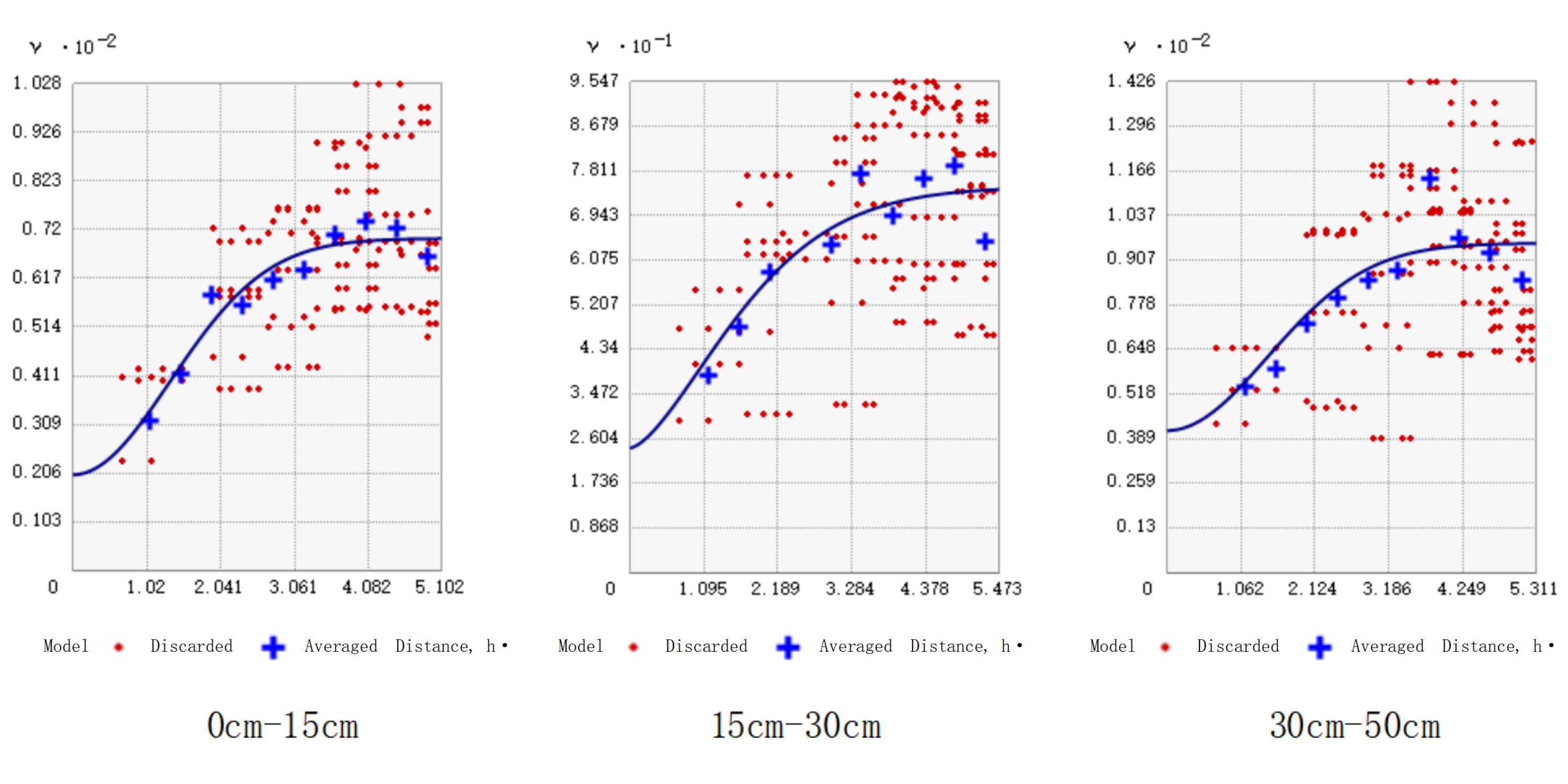

Supplement: Supplemental Information 9 [file peerj-12-18724-s009.png]

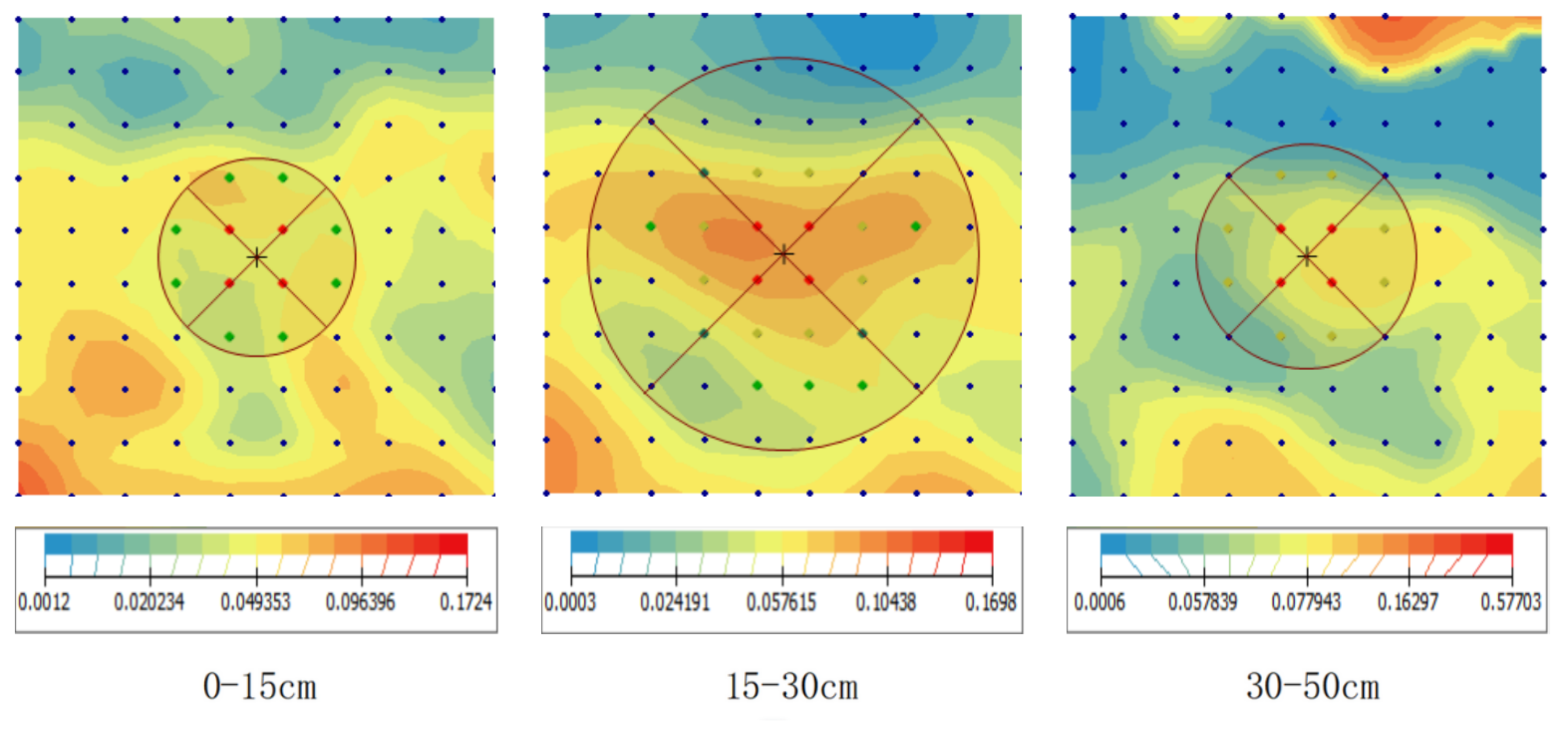

Supplement: Supplemental Information 10 [file peerj-12-18724-s010.png]

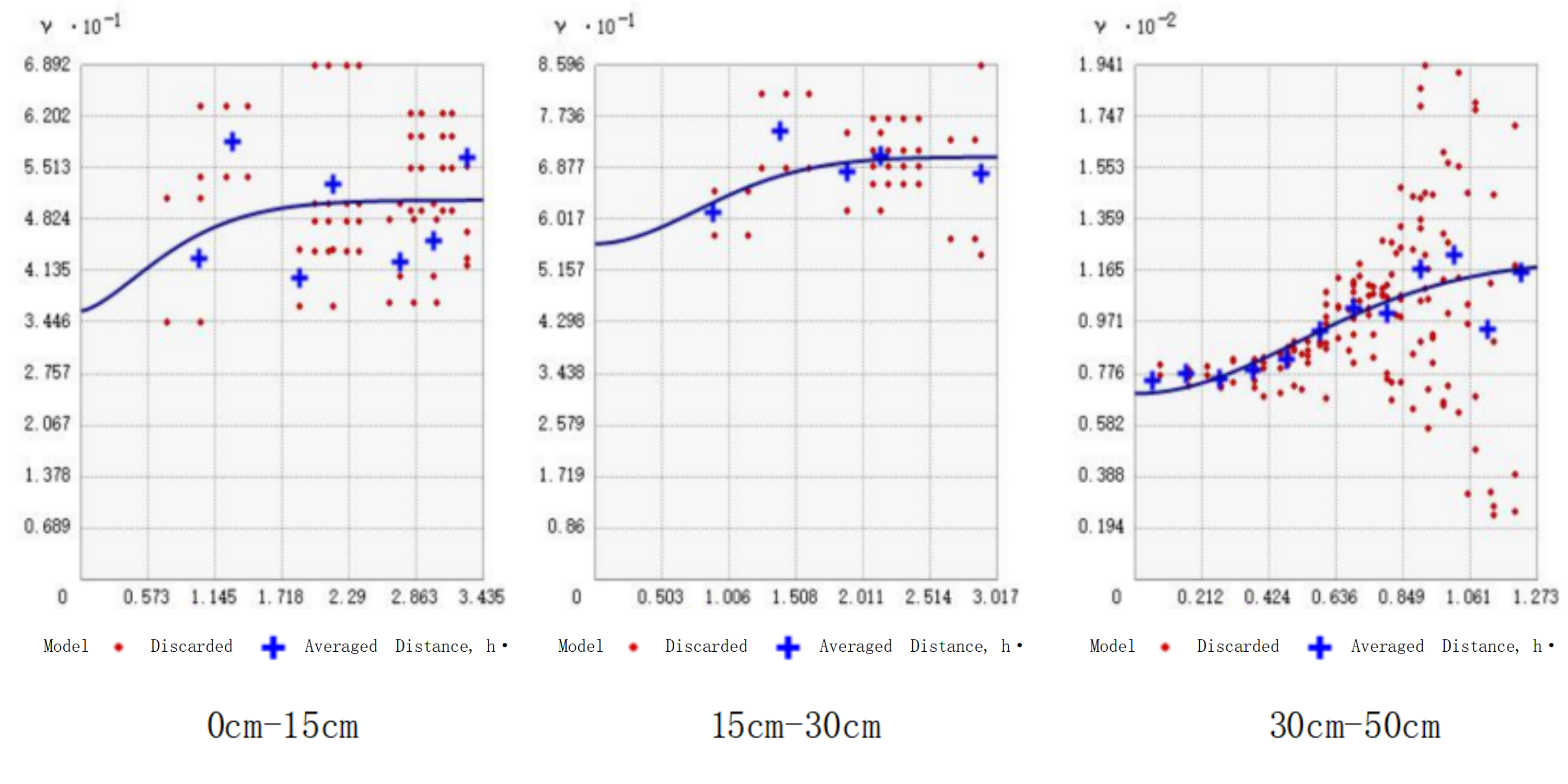

Supplement: Supplemental Information 11 [file peerj-12-18724-s011.png]

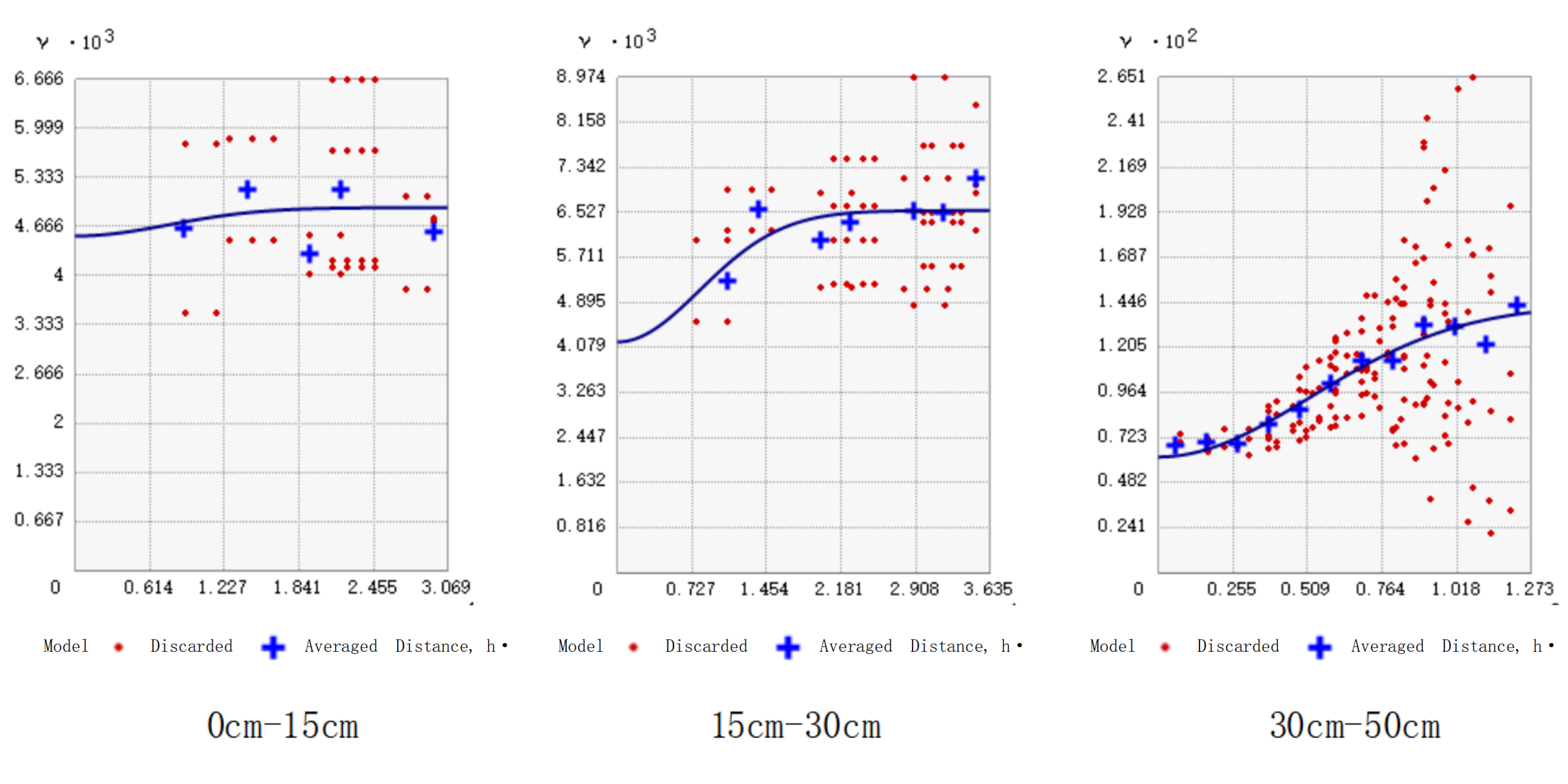

Supplement: Supplemental Information 12 [file peerj-12-18724-s012.png]

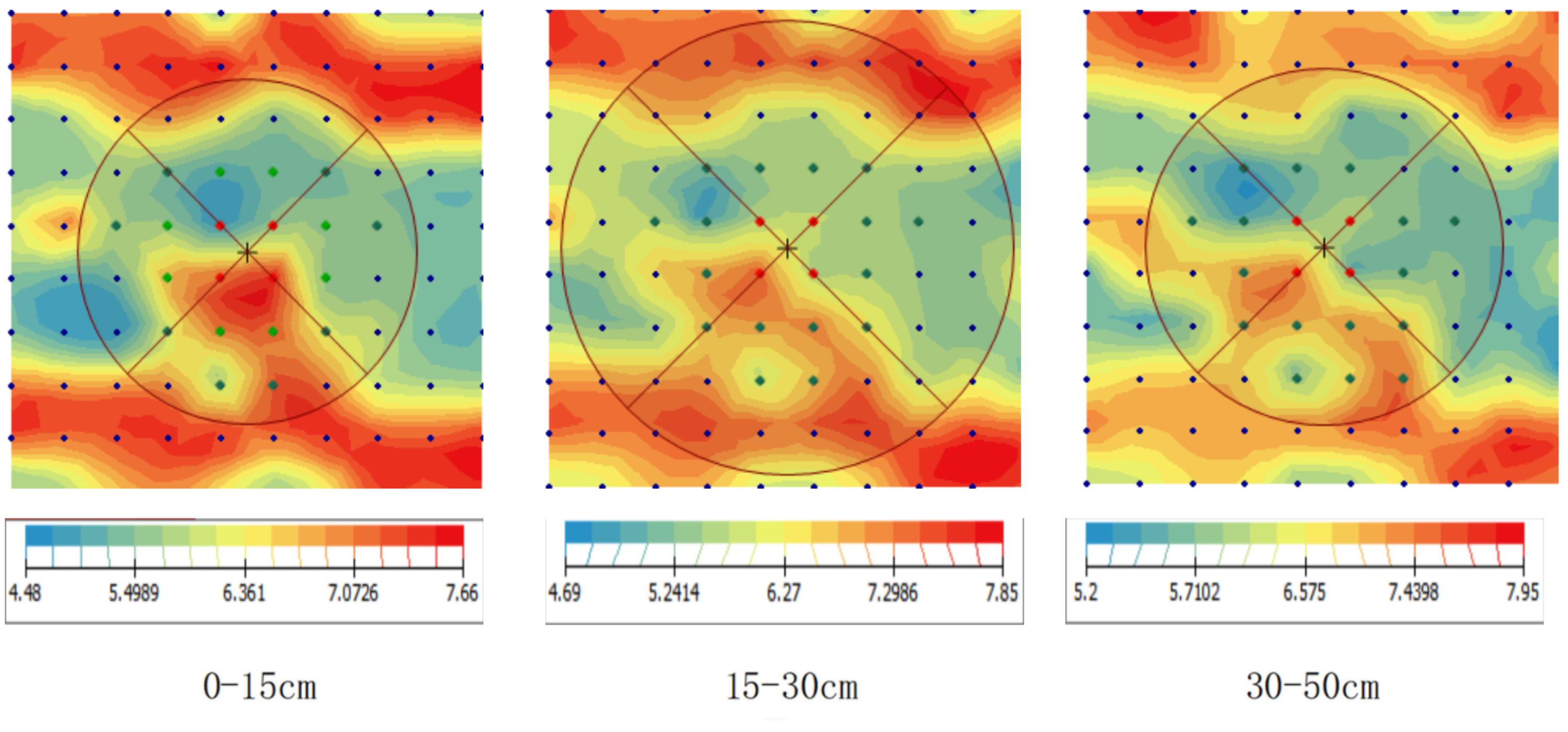

Supplement: Supplemental Information 13 [file peerj-12-18724-s013.png]

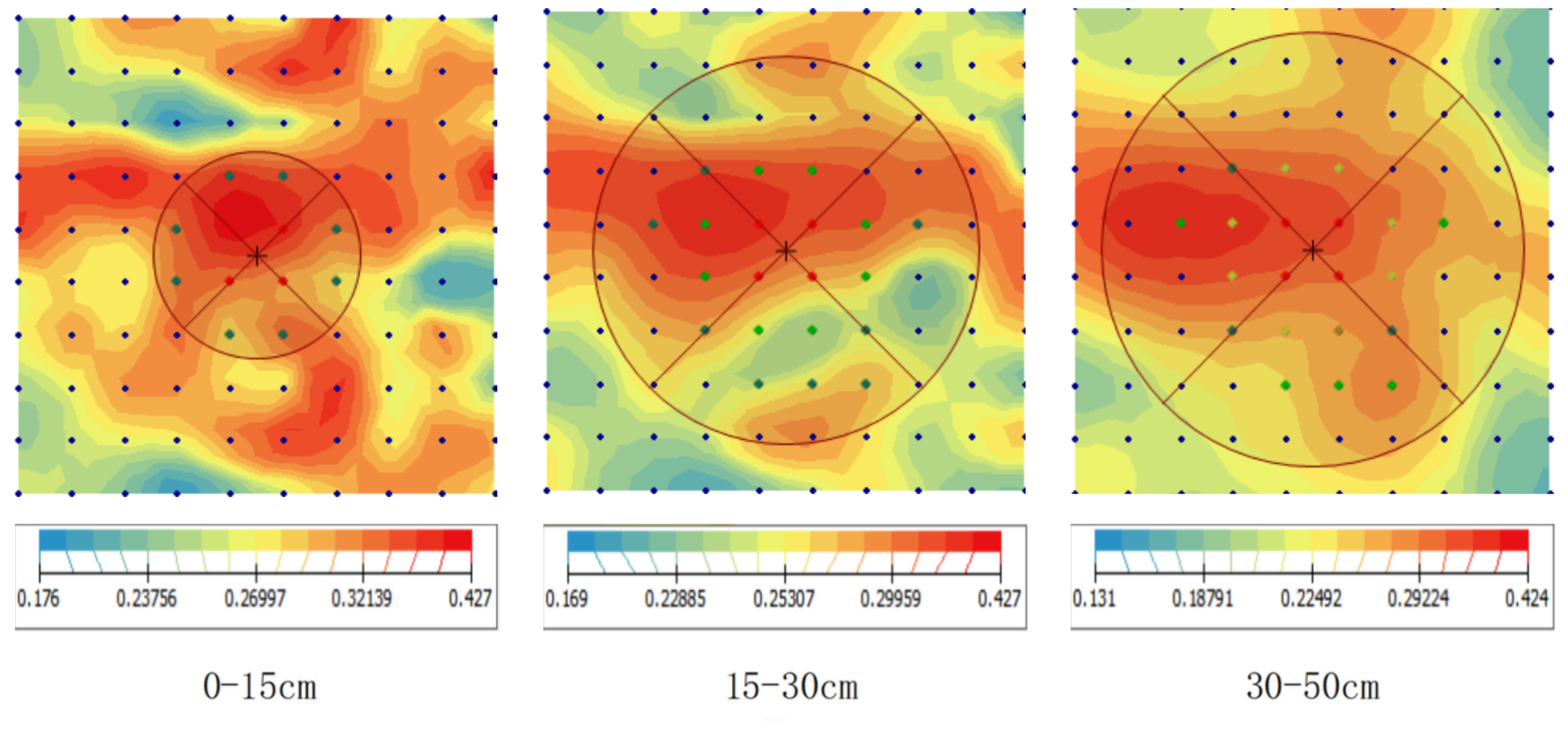

Supplement: Supplemental Information 14 [file peerj-12-18724-s014.png]

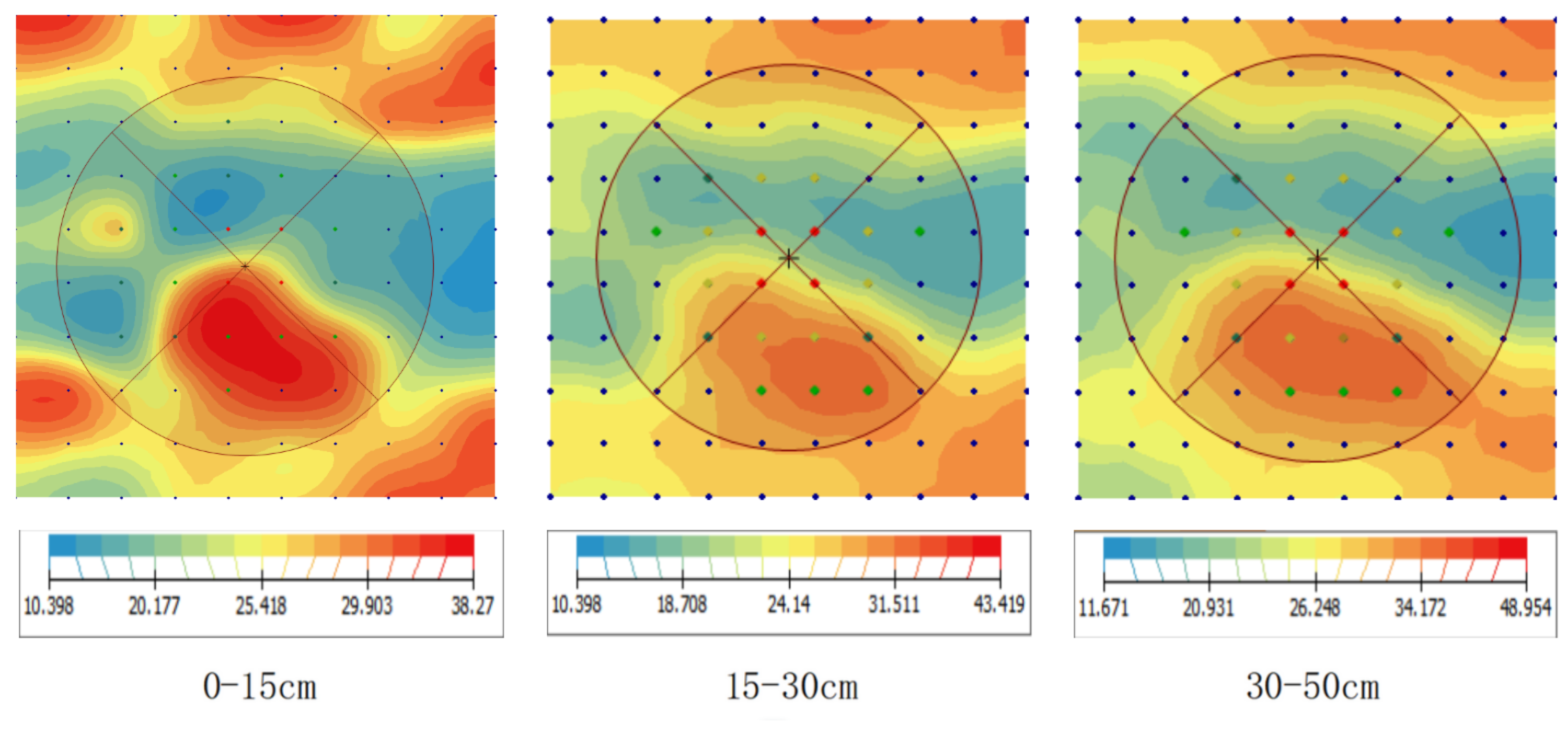

Supplement: Supplemental Information 15 [file peerj-12-18724-s015.png]

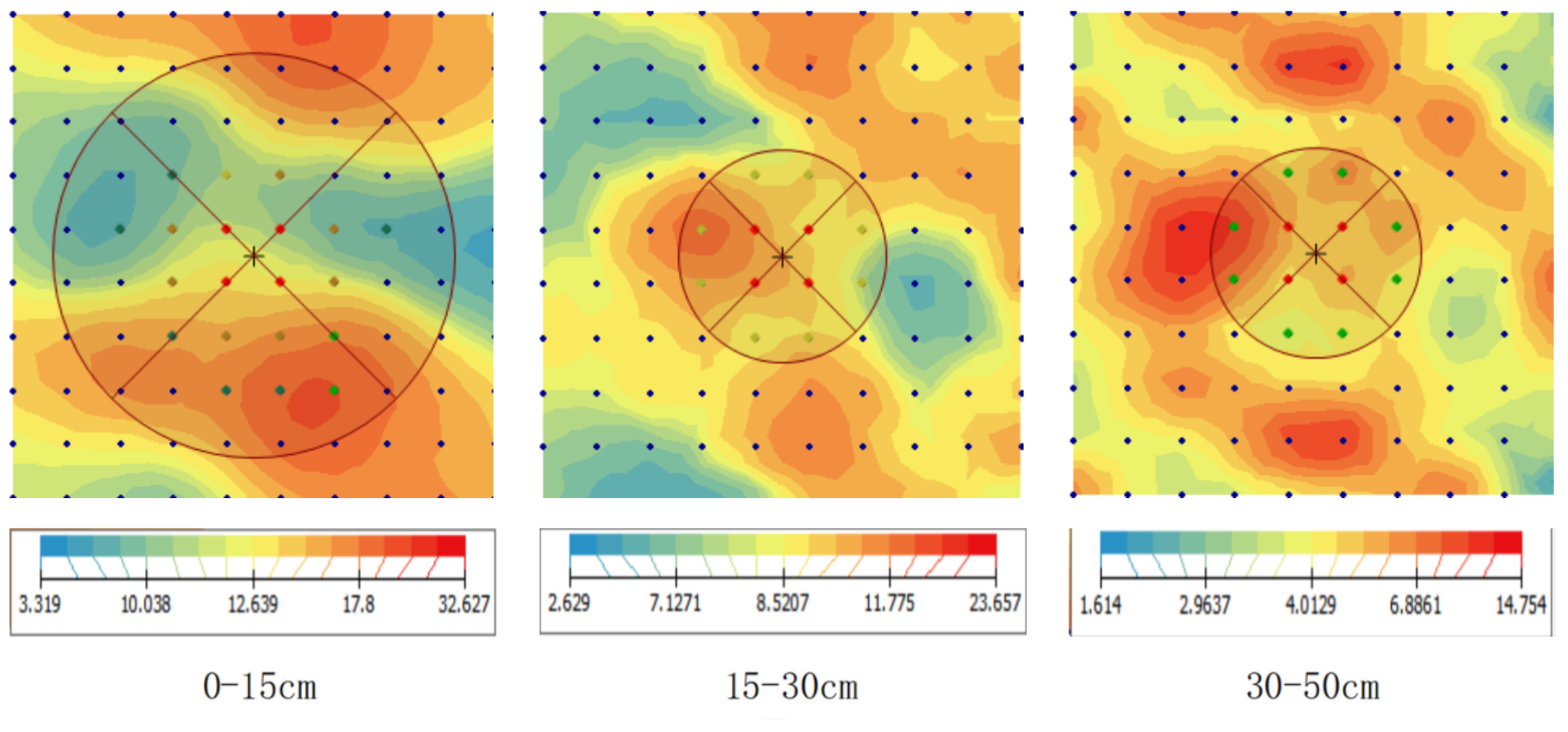

Supplement: Supplemental Information 16 [file peerj-12-18724-s016.png]

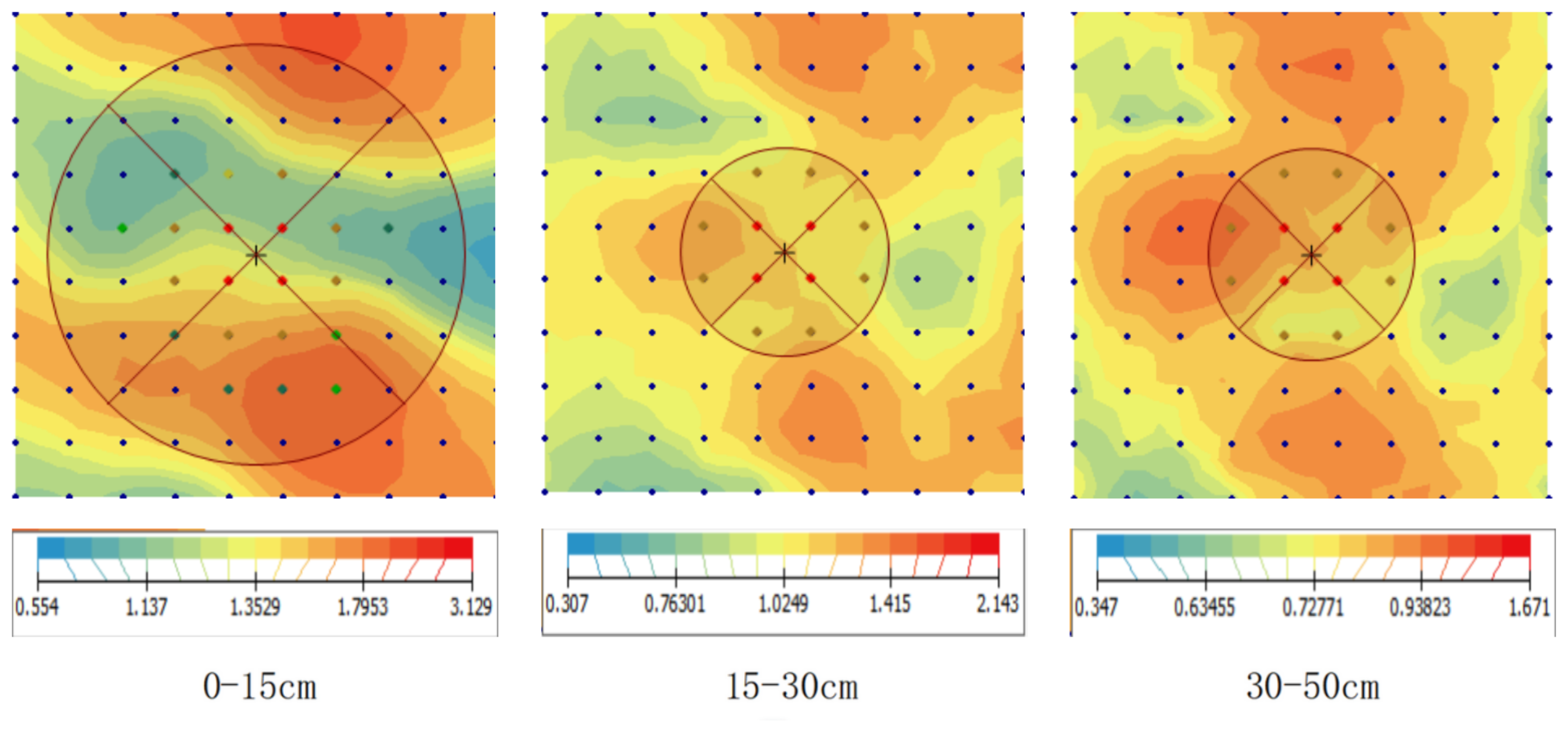

Supplement: Supplemental Information 17 [file peerj-12-18724-s017.png]
